# Dydrogesterone and Letrozole Combination for the Treatment of Endometriosis: A Mechanism‐Based Therapeutic Approach

**DOI:** 10.1155/ogi/5464578

**Published:** 2026-02-13

**Authors:** Santosh Kumar Rai, Rakesh Kumar, Mohd Imran Khan, Anil Kumar

**Affiliations:** ^1^ New Drug Discovery Research, Mankind Research Centre, Mankind Pharma Limited, 122051, Gurugram, India

**Keywords:** combination, dydrogesterone, endometriosis, letrozole

## Abstract

Endometriosis is a complex, estrogen‐dependent disease with limited effective treatments that often focus on symptom management rather than addressing the underlying pathology. Current therapies, such as progestins and GnRH agonists, have significant side effects (weight gain, mood changes, decreased bone mineral density, and menopausal symptoms) and fail to prevent disease recurrence or address fertility concerns. The current study is the first to demonstrate the therapeutic potential of combined treatment with dydrogesterone (a progestin) and letrozole (an aromatase inhibitor) in a preclinical mouse model of endometriosis. Our results revealed that the dydrogesterone–letrozole combination reduced the volume of endometriotic lesions, suppressed cell proliferation, and decreased inflammation relative to the disease control, with trends suggesting greater effects than those observed with individual agents or standard treatments such as dienogest and leuprolide. The dydrogesterone–letrozole combination also exhibited a reduction in fibrosis, indicating a potential role in managing chronic endometriosis and associated symptoms such as pelvic pain and adhesions. These findings suggest that the dydrogesterone–letrozole combination may offer a broader therapeutic approach for endometriosis. Based on existing literature, dydrogesterone is thought to counteract estrogen‐driven endometrial proliferation through progesterone receptor activation, while letrozole is believed to reduce estrogen biosynthesis by inhibiting aromatase, which may collectively influence lesion growth and inflammatory processes. Additional long‐term studies are warranted to thoroughly assess the safety profile, clinical effectiveness, and overall therapeutic relevance of the dydrogesterone–letrozole combination for the treatment of endometriosis.

## 1. Introduction

Endometriosis is a chronic, estrogen‐dependent, and often debilitating gynecological disorder affecting approximately 10% of women of reproductive age, leading to severe pelvic pain, infertility, and a substantial decline in quality of life [1, 2]. It is characterized by the presence of endometrial‐like tissue outside the uterine cavity, which provokes chronic inflammation, fibrosis, and the formation of ectopic lesions. Abnormalities observed in endometriosis include altered uterine anatomy, dysregulated reproductive function, elevated local estrogen synthesis, abnormal endometrial cell proliferation, and ectopic expression of aromatase in endometrial tissue [1, 3].

Aromatase, the key enzyme responsible for converting androgens to estrogens, is not typically expressed in the healthy endometrium but is aberrantly overexpressed in endometriotic lesions. This leads to localized estrogen biosynthesis, which promotes the survival, proliferation, and inflammatory activity of ectopic endometrial cells, creating a self‐sustaining loop that contributes to disease persistence and progression [4]. Estrogen further promotes the production of vascular endothelial growth factor (VEGF), which enhances angiogenesis and the epithelial‐to‐mesenchymal transition (EMT), contributing to lesion expansion and invasion [1, 5].

Despite the high global prevalence and clinical burden of endometriosis, current therapeutic strategies are largely symptomatic and fail to address the underlying pathophysiology. Hormonal therapies—including progestins, combined oral contraceptives (COCs), gonadotropin‐releasing hormone (GnRH) agonists, GnRH antagonists—as well as surgical interventions, are commonly employed [6].

Progestins such as dienogest, medroxyprogesterone acetate (MPA), and norethisterone are widely used due to their ability to inhibit endometrial proliferation, reduce menstrual bleeding, and alleviate pain. However, their efficacy is limited in severe endometriosis, and long‐term use is often associated with side effects such as irregular bleeding, weight gain, and mood changes [7]. Moreover, endometriosis is often associated with infertility [1], and the effect of progestin therapy on fertility is still an important concern, since dienogest, medroxyprogesterone, and danazol could not improve fertility in women of reproductive age [8, 9].

In this context, another progestin, dydrogesterone, presents a promising alternative with a more favorable therapeutic profile [10]. Dydrogesterone closely resembles natural progesterone and exhibits antiproliferative effects by inducing decidualization and atrophy of ectopic endometrial lesions without affecting the fertility. Its high selectivity for progesterone receptors, without significant androgenic, estrogenic, or glucocorticoid activity, contributes to a lower incidence of side effects commonly seen with other progestins like dienogest [11]. This targeted action of dydrogesterone leads to effective endometrial suppression without inhibiting ovulation, making it a suitable option for women who want to get pregnant [10]. Although dydrogesterone has shown a favorable therapeutic and safety profile, its efficacy remains limited in suppressing local estrogen (E2) production, which is a key factor in the progression of endometriosis [12].

The aromatase enzyme mediates the conversion of androstenedione and testosterone to estrone and E2 [1, 13]. Among the different aromatase inhibitors, letrozole has demonstrated the most effective suppression of estrogen [14]. The reduction in estrogen levels by letrozole has been reported to suppress the growth of ectopic endometrial tissue, alleviate pain, and reduce inflammation [14]. Letrozole also prevents the recurrence of lesions and improves fertility outcomes in women with endometriosis‐related infertility. Previous studies showed that a long‐term administration of aromatase inhibitors including letrozole (6 months or more) is associated with several adverse effects (osteoporosis, hot flashes, headache, and joint pain etc.). However, letrozole in combination with progestins effectively managed the adverse events by balancing the hypoestrogenic effects and demonstrated higher efficacy in patients with endometriosis [15–17].

In the current study, we have evaluated the effect of the dydrogesterone–letrozole combination in a preclinical mouse model of endometriosis. Our results demonstrated that the dydrogesterone–letrozole combination was associated with reductions in disease‐related parameters relative to the disease control, with trends indicating greater effects than those observed with monotherapies (dydrogesterone or letrozole alone) and standard treatments such as leuprolide and dienogest; however, no direct statistical comparisons between treatment groups were performed. Moreover, a study conducted in human hepatocytes indicated no evidence of drug–drug interactions between dydrogesterone and letrozole. Overall, this study provides the first preclinical insight into the combined effects of dydrogesterone and letrozole, laying the groundwork for future translational research and therapeutic development.

## 2. Materials and Methods

### 2.1. Drug–Drug Interaction Assay

1 × 10^6^ human hepatocyte cells/mL (> 95% viability) were added to individual wells in 24‐well plates and incubated in a CO_2_ incubator at 37°C and 5% CO_2_ for 15 min. Zero‐min incubation was terminated by adding 500 μL of ice‐cold acetonitrile containing 0.45 μm lansoprazole. All the reactions were initiated by adding 250 μL of test compounds diluted in KHB (prewarmed at 37°C in a CO_2_ incubator) and further incubated for 0, 30, 60, and 90 min in a CO_2_ incubator with gentle shaking. At the end of each time point, reactions were terminated by adding 500 μL of ice‐cold acetonitrile, and samples were analyzed by LC–MS/MS to calculate the half‐life and intrinsic clearance.

### 2.2. Animals

Female BALB/c mice of age 7–8 weeks were used for the study and maintained in accordance with the Committee for Control and Supervision of Experiments on Animals (CCSEA, India). All the animals were allowed to quarantine for 3 days and acclimatize for one day prior to surgery. During this period, the mice were observed daily for clinical signs, general activity, and any signs of distress. Mice were maintained in a barrier unit in a well‐controlled, pathogen‐free environment with regulated cycles of 12 h light/12 h dark (22°C–25°C). Mice had free access to food and water. All procedures of the present study were performed according to the guidelines provided by the CCSEA.

Animals were further monitored daily throughout the postoperative and treatment periods for clinical signs, morbidity, mortality, and welfare indicators. Body weights were recorded at regular intervals. No adverse events or animal deaths occurred during the study. Humane endpoints (e.g., > 15% body‐weight loss, impaired mobility, or signs of pain/distress unresponsive to analgesia) were predefined, and no animals reached these criteria.

### 2.3. Surgery

We induced endometriosis‐like lesions through transplantation of one of the uterine horns to the peritoneal cavity, as described previously [18]. All procedures in the present study were performed in accordance with the guidelines provided by the CCSEA as published in the Gazette of India, December 15, 1998. Prior approval of the Institutional Animal Ethics Committee (IAEC) was obtained (IAEC Proposal No.: FB‐24‐04). Mice with estrous cycles were anesthetized using isoflurane anesthesia (3%–3.5% induction, 1.5%–2% maintenance), and oophorectomy was performed. After the oophorectomy, uterine horns were cut longitudinally into small 2‐mm pieces and anchored onto the abdominal wall of the same mice using sterile 6‐0 surgical sutures. The abdominal wall and skin were closed using 6‐0 silk surgical sutures. Appropriate postoperative care (povidone–iodine solution and antibiotic) and pain management (meloxicam administered as perioperative analgesia, with additional postoperative doses as required based on clinical monitoring) were performed after the surgery. Animals were closely monitored during recovery until full ambulation was regained and then observed once daily for surgical site integrity, pain‐related behaviors, and general welfare. Exogenous estradiol (10 mg/kg in sesame oil) was administered subcutaneously twice weekly, as high‐dose estrogen supplementation is widely employed in murine endometriosis models to maintain a sustained estrogen‐dominant environment that supports consistent lesion establishment, angiogenesis, and fibrosis. Prior studies have demonstrated that supraphysiological estradiol enhances pathological processes relevant to endometriosis, including the upregulation of adhesion and fibrosis‐promoting factors [18–20]. A fat pad was implanted onto the peritoneum to create sham control. Endometrial implants were allowed to grow for 14 days.

### 2.4. Animal Treatment

On 15th day (Day 1 of treatment), animals were randomized into different groups based on body weight and treated for 28 days. Group sizes (*n* = 6–8 per group) were selected based on previously published studies using similar animal models and outcome measures, which showed that this range was sufficient to detect meaningful biological effects in preclinical settings [18, 19, 21]. The study groups comprised sham control (G1); vehicle/disease control (G2); dydrogesterone (5 mg/kg) and letrozole (0.5 mg/kg) (G3); dydrogesterone alone (5 mg/kg) (G4); letrozole alone (0.5 mg/kg) (G5); dienogest (0.3 mg/kg) (G6); and leuprolide (1 mg/kg) (G7). Groups G2–G6 were orally administered with respective treatments once daily for 28 days. A single dose of leuprolide (1 mg/kg) was administered subcutaneously to G7 with a dosing volume of 5 mL/kg. Naïve control and vehicle control received 10 mL/kg of vehicle (0.5% v/v Tween‐80 and 99.5% methylcellulose [0.5% w/v in RO water]). The dosing volume of each formulation was kept constant at 10 mL/kg to all mice in G2–G6 throughout the dosing period. The dosage of each medication for the mice was based on the FDA‐approved clinical dose for human and then converted to animal dose according to the guidelines of the FDA calculator based on the surface area of humans and experimental animals [22, 23]. Body weights of all animals were recorded at the time of randomization and twice weekly prior to dosing. At the end of the study (on 28^th^ day of treatment), blood was collected from all groups G1 to G7 and used for estimation of biomarkers by ELISA. Endometriosis lesion volume of each animal was measured, and lesions were also analyzed for histopathology and IHC (proliferating cell nuclear antigen [PCNA]).

### 2.5. Evaluation of Ectopic Uterine Tissue

After 28 days of treatment, animals were humanely sacrificed by carbon dioxide asphyxiation. Endometriotic lesions were measured in two perpendicular diameters using a Vernier caliper. The volume of each ectopic uterine tissue was calculated by the following formula: *V* = (4/3) π $r_{1}^{2}$ *r*_2_, where *r*_1_ and *r*_2_ are the radii and *r*_1_ < *r*_2_ [24].

### 2.6. Histopathology

Histology was performed for endometriosis lesions using hematoxylin and eosin (H&E) staining. Briefly, specimen tissue from all the experimental groups was fixed in 10% buffered neutral formalin for one week at room temperature. Then, tissues were dehydrated in a graded series of alcohol, cleaned in xylene, and then embedded in paraffin. Serial sections of 4‐mm thickness were prepared from each tissue‐embedded paraffin blocks using a rotary microtome and examined using a light microscope after H&E staining. Blind histopathological evaluation of endometriosis tissue was performed by the pathologist. Fibrosis in the sections was assessed and graded on a scale of 1 to 4 (HPF). A grading scale (0–3) was used to determine the histopathological score and sample tissue epithelial proliferation.

### 2.7. Immunohistochemistry

For immunostaining, paraffin wax–embedded tissue blocks were sectioned at 4–6 μm thickness with the rotary microtome and placed on slides coated with poly‐L‐lysine and incubated overnight at 37°C for 1 h. Further, these sections were stained with PCNA (PC10) primary antibodies (Mouse mAb #2586) and developed with peroxidase‐labeled goat antirabbit IgG as a secondary antibody. The staining was visualized by reaction with diaminobenzidine color reagent and then counterstained with hematoxylin. Finally, the sections were rinsed with tris buffer saline (TBS) and dehydrated in alcohol and cleared in xylene prior to mounting using DPX. All the sections were examined under a light microscope to record the intensity of the antigen–antibody reaction.

### 2.8. Quantification of Cytokines

After 4 weeks of treatment, animals were sacrificed by carbon dioxide asphyxiation, and blood was collected from all groups (G1–G7) under isoflurane. Blood was allowed to clot for 15–20 min, and the serum samples were separated by centrifugation at 4°C, 5000 RPM for 10 min. Serum samples collected were further used for the ELISA/LEGENDplex ELISA assays for the estimation of TNF‐α, IL‐6, IL‐1β, and VEGF. Endometriotic tissues collected were also extracted for the estimation of TNF‐α, IL‐6, IL‐1β, and VEGF.

### 2.9. Statistical Analysis

Results are shown as means SD or SEM. GraphPad Prism Version 5.01 was used for statistical analysis. Statistical comparisons between two groups were made using Student’s *t*‐test. Comparisons between more than two groups were made by one‐way analysis of variance (ANOVA), followed by Dunnett’s test. *p* values of < 0.05 were considered significant.

## 3. Results

### 3.1. Drug–Drug Interaction of Dydrogesterone and Letrozole in Human Hepatocytes

We evaluated the potential drug–drug interaction between dydrogesterone and letrozole using human hepatocytes. Dydrogesterone is primarily metabolized in the liver by AKR1C1, with minor involvement from liver enzymes CYP3A4 and CYP2C19 [25], while letrozole is mainly metabolized by CYP2A6, with a smaller contribution from CYP3A4 [26]. The results showed that the metabolism of dydrogesterone and letrozole remained unchanged in the dydrogesterone–letrozole combination, indicating no metabolic interference (Table 1).

**TABLE 1:** Drug–drug interaction analysis between dydrogesterone and letrozole in human hepatocytes.

Compound	Human hepatocytes	
	T_1/2_ (in min)	Clearance (μL/min)
Dydrogesterone alone	31.50	44
Dydrogesterone (+letrozole)	21.66	64
Letrozole alone	> 120	1.5
Letrozole (+dydrogesterone)	> 120	1.5

### 3.2. Effects of Dydrogesterone–Letrozole Combination on Ectopic Uterine Tissue Growth

In our study, endometriotic lesions were implanted in all Balb/c mice by surgery (disease control and treatment groups) and allowed to grow for 2 weeks. Endometriosis was successfully developed in all mice without any mortality. Thereafter, animals were treated with their respective treatments (Materials and Methods, Sections 2.3 and 2.4) for 28 days.

**FIGURE 1:**
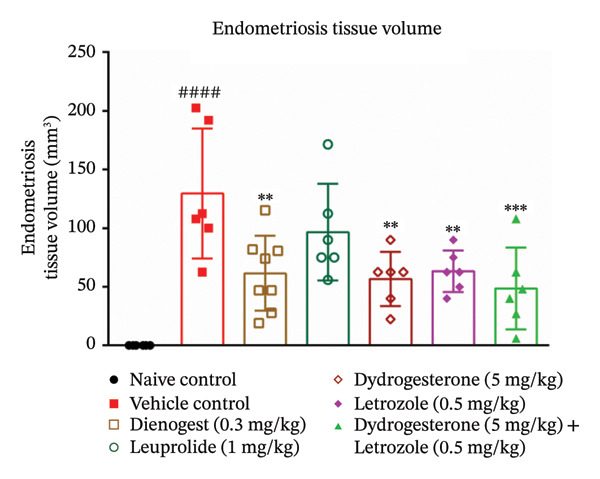
Effect of the dydrogesterone–letrozole combination on the endometriotic lesion volume in a surgically induced endometriosis mouse model. One‐way ANOVA followed by Dunnett’s multiple comparison test was used for statistical analysis. Data are shown as mean ± S.E.M. (*n* = 6–8), ^#^Significant difference as compared to the naive control group. ^∗^Significant difference as compared to the disease/vehicle control group. ^#/∗^ *p* < 0.05, ^##/∗∗^ *p* < 0.01, ^###/∗∗∗^ *p* < 0.001, and ^####/∗∗∗∗^ *p* < 0.0001.

**FIGURE 2:**
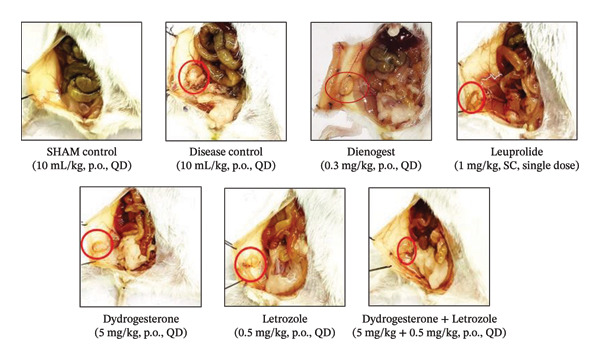
Representative images of endometriotic tissue growth in treatment and nontreatment groups.

At the end of the treatment phase, the volume of endometriotic lesions was measured using a Vernier caliper. A significant increase in lesion volume was observed in the disease control group (Figures 1 and 2). Treatment with the dydrogesterone–letrozole combination was associated with a reduction in lesion volume compared to the disease control, with trends indicating greater effects than those observed with either monotherapy.

Among the reference drugs, leuprolide did not produce a statistically significant decrease in lesion volume compared to the disease control group, while dienogest was associated with reduced lesion volume, with a trend that appeared less pronounced than that of the dydrogesterone–letrozole combination (Figure 1). All treatment groups were statistically evaluated relative to the disease control; however, direct pairwise comparisons among treatment groups were not conducted.

### 3.3. Effects of Dydrogesterone–Letrozole Combination on Histopathological Parameters of Endometriotic Tissue

The dydrogesterone–letrozole combination was further evaluated for its effect on the total histopathological score (Figure 3(d)), which included assessments of stromal tissue (Figure 3(b)), glandular tissue (Figure 3(c)), epithelial proliferation (Figure 3(a)), and fibrosis (Figure 3).

FIGURE 3Effect of the dydrogesterone–letrozole combination on the histological markers. Epithelial proliferation (a), stromal tissue score (b), glandular tissue score (c), and total histopathology score (d) in a surgically induced endometriosis mice model. One‐way ANOVA followed by Dunnett’s multiple comparison test was used for statistical analysis. Data are shown as mean ± S.E.M. (*n* = 6–8). ^#^Significant difference as compared to the naive control group. ^∗^Significant difference as compared to the disease/vehicle control group. ^#/∗^ *p* < 0.05, ^##/∗∗^ *p* < 0.01, ^###/∗∗∗^ *p* < 0.001, and ^####/∗∗∗∗^ *p* < 0.0001.

**Figure:**
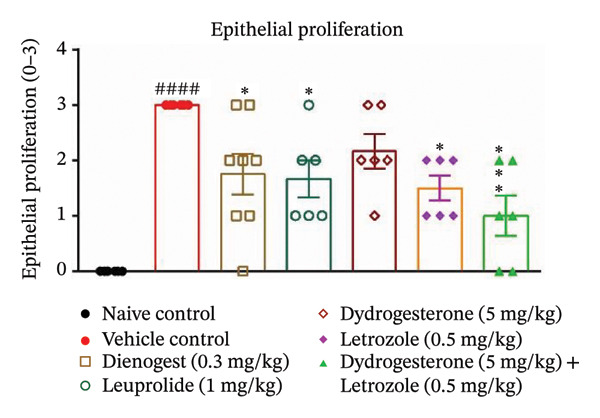
(a)

**Figure:**
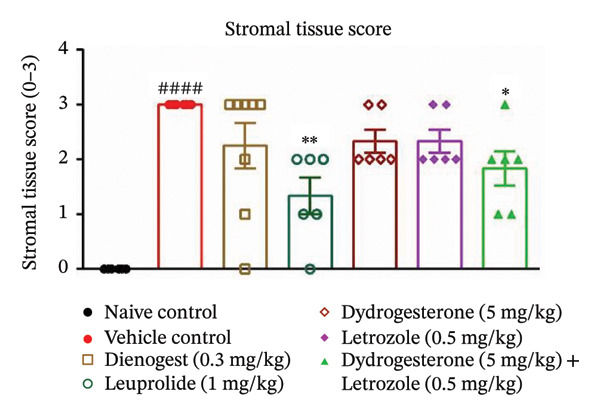
(b)

**Figure:**
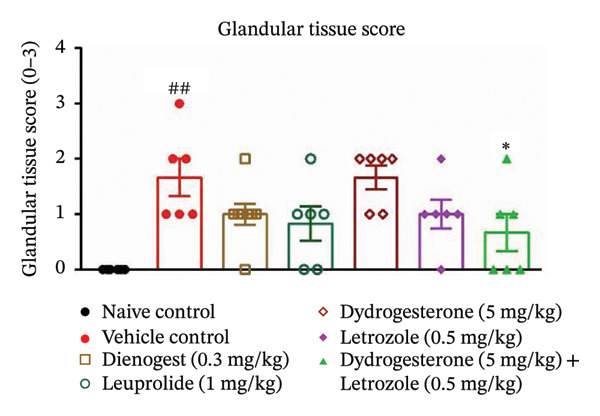
(c)

**Figure:**
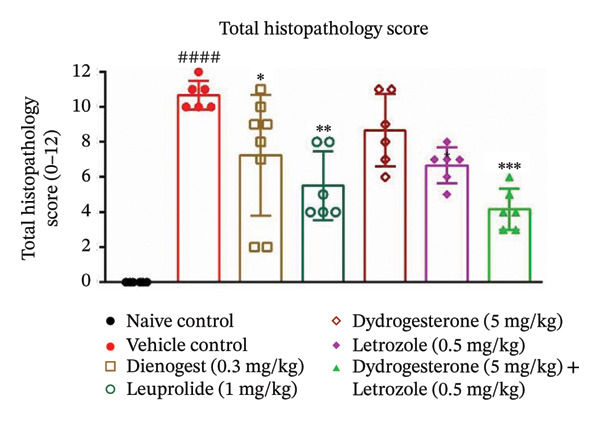
(d)

The dydrogesterone–letrozole combination led to a noticeable decrease in individual histopathological parameters, including stromal tissue score, glandular tissue score, fibrosis score, and epithelial proliferation score relative to the disease control group (Figures 3(a), 3(b), 3(c)). These reductions indicate an overall improvement in histopathological features compared with disease control. Consequently, the cumulative effect of these improvements was reflected in a significant decrease in the total histopathology score with respect to the disease control group (Figure 3(d)). In contrast, treatment with dydrogesterone alone did not result in a significant decline across all histopathological parameters when compared to the disease control group, whereas letrozole monotherapy primarily inhibited epithelial proliferation without substantially affecting other parameters (Figure 3(a)). These observations suggest that individual agents exhibited more limited effects on certain histopathological features under the conditions tested. Similarly, standard drugs dienogest and leuprolide could not significantly inhibit all histopathological parameters compared to the vehicle control. The dydrogesterone–letrozole combination was associated with a reduction in total histopathology score relative to the disease control, with trends suggesting effects beyond those observed with monotherapies and reference treatments; however, no direct statistical comparisons between treatment groups were performed (Figures 3(a), 3(b), 3(c), and 3(d)).

### 3.4. Effect of Dydrogesterone–Letrozole Combination on Cell Proliferation in Ectopic Uterine Tissue

**FIGURE 4:**
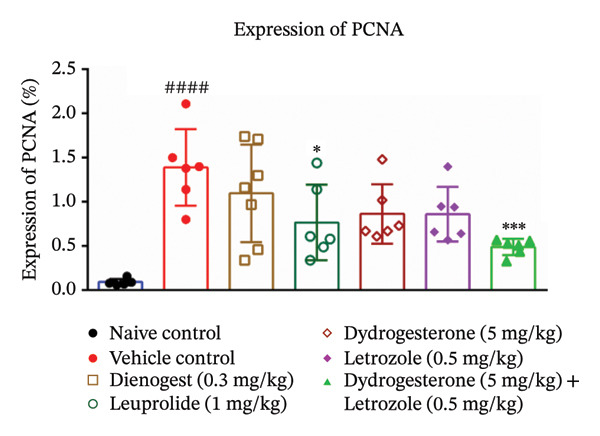
Effect of the dydrogesterone–letrozole combination on cell proliferation of endometriosis tissue. One‐way ANOVA followed by Dunnett’s multiple comparison test was used for statistical analysis. Data are shown as mean ± S.E.M. (*n* = 6–8). ^#^Significant difference as compared to the sham control group. ^∗^Significant difference as compared to the disease/vehicle control group. ^#/∗^ *p* < 0.05, ^##/∗∗^ *p* < 0.01, ^###/∗∗∗^ *p* < 0.001, and ^####/∗∗∗∗^ *p* < 0.0001.

**FIGURE 5:**
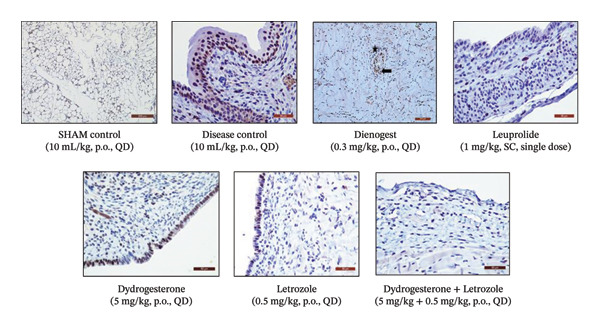
PCNA expression in endometriotic tissues from control and treated mice. PCNA expression was evaluated by immunohistochemistry using a polyclonal antibody.

We further investigated the antiproliferative effects of the dydrogesterone–letrozole combination on endometriotic lesions through immunohistochemical staining of PCNA, a well‐established marker for cellular proliferation. An elevated number of PCNA‐positive cells was observed in the vehicle‐treated disease control group, indicating enhanced cellular proliferation associated with active endometriotic lesions. Treatment with the dydrogesterone‐–etrozole combination was associated with a reduction in PCNA‐positive cells relative to the vehicle control group, suggesting a trend toward suppression of aberrant cell proliferation. In contrast, treatment with dydrogesterone alone or letrozole alone produced only modest suppression in PCNA expression, indicating less pronounced antiproliferative trends when used as monotherapies (Figures 4 and 5). Overall, the data suggest that the dydrogesterone–letrozole combination may be associated with greater attenuation of proliferative activity within endometriotic tissue relative to individual agents; however, direct statistical comparisons between treatment groups were not performed, and these observations should therefore be interpreted as descriptive trends (Figures 4 and 5). Among the reference treatments, leuprolide significantly inhibited PCNA expression compared with the disease control group, consistent with its known clinical role in suppressing estrogen‐driven proliferation [27]. Dienogest, however, did not significantly reduce the number of PCNA‐positive cells, suggesting limited antiproliferative activity under the experimental conditions employed in this study.

### 3.5. Effect of Dydrogesterone–Letrozole Combination on Inflammation and Fibrosis in Endometriotic Mice

To investigate the anti‐inflammatory effects of the dydrogesterone–letrozole combination in endometriotic mice, levels of VEGF and key proinflammatory cytokines were assessed in excised endometriotic lesions. The vehicle‐treated disease control group exhibited elevated levels of VEGF, IL‐6, TNF‐α, and IL‐1β compared to the naïve control group, indicating an enhanced inflammatory and angiogenic response associated with endometriosis progression.

Treatment with the dydrogesterone–letrozole combination was associated with reductions in VEGF (Figure 6(a)), TNF‐α (Figure 6(b)), and IL‐1β (Figure 6(c)) levels in the endometriotic lesions relative to the disease control group, suggesting a trend toward modulation of inflammatory and angiogenic markers. IL‐6 levels also declined, although this decrease did not reach statistical significance relative to the disease control (Figure 6(d)). Monotherapies with dydrogesterone or letrozole, as well as the reference drugs leuprolide and dienogest, produced modest or nonsignificant changes in these inflammatory markers. Overall, the data suggest that the dydrogesterone–letrozole combination may be associated with greater modulation of inflammatory and angiogenic mediators relative to the disease control, with trends indicating effects beyond those observed with other treatments (Figure 6).

FIGURE 6Effect of the dydrogesterone–letrozole combination on VEGF and inflammatory factors in endometriotic lesions of mice. The tissue levels of VEGF (a), TNF‐α (b), IL‐1β (c), and IL‐6 (d). One‐way ANOVA followed by Dunnett’s multiple comparison test was used for statistical analysis. Data are shown as mean ± S.E.M. (*n* = 6–8). ^#^Significant difference as compared to the sham control group. ^∗^Significant difference as compared to the disease/vehicle control group. ^#/∗^ *p* < 0.05, ^##/∗∗^ *p* < 0.01.

**Figure:**
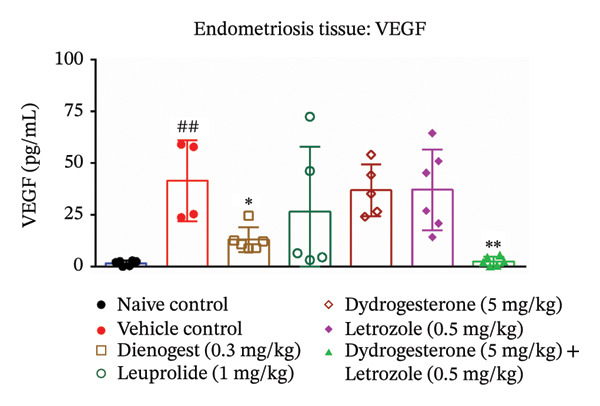
(a)

**Figure:**
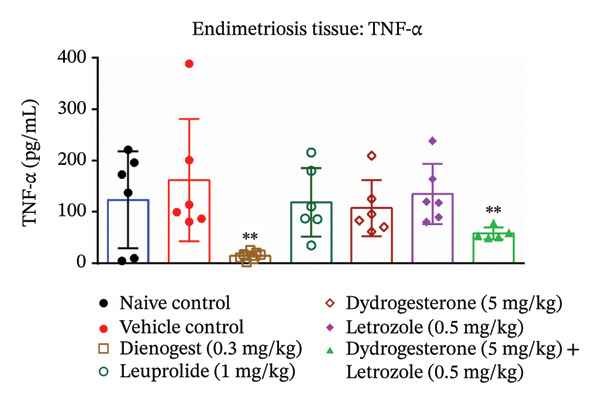
(b)

**Figure:**
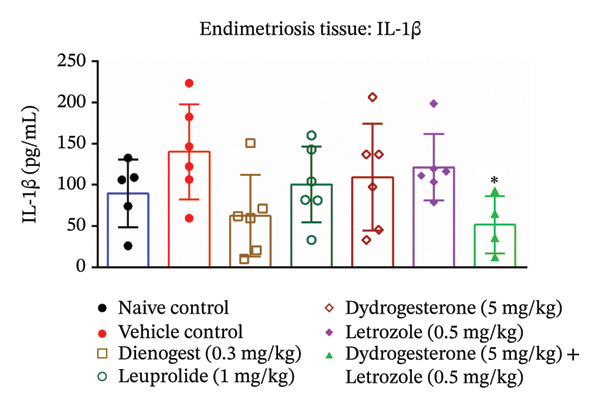
(c)

**Figure:**
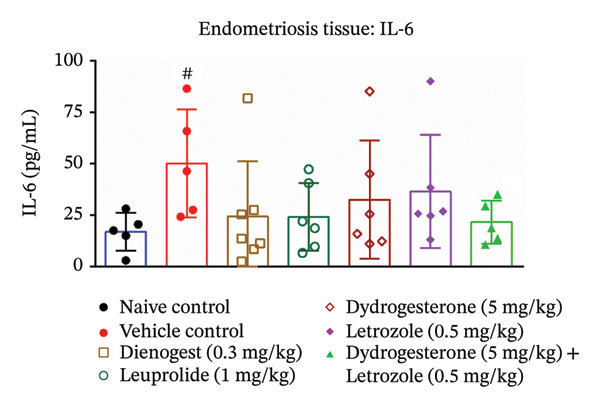
(d)

**FIGURE 7:**
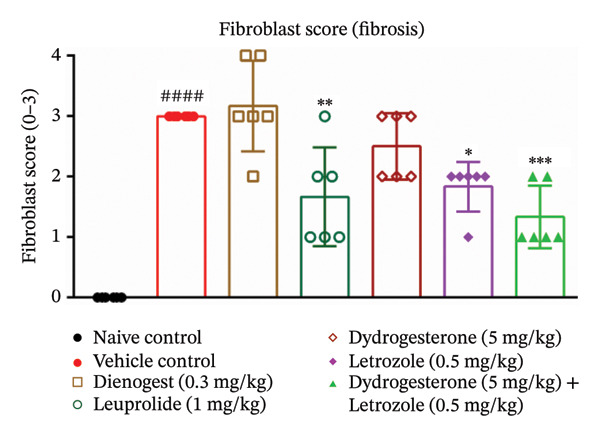
Effect of the dydrogesterone–letrozole combination on fibrosis in endometriotic lesions of mice. One‐way ANOVA followed by Dunnett’s multiple comparison test was used for statistical analysis. Data are shown as mean ± S.E.M. (*n* = 6–8). ^#^Significant difference as compared to the sham control group. ^∗^Significant difference as compared to the disease/vehicle control group. ^#/∗^ *p* < 0.05, ^##/∗∗^ *p* < 0.01, ^###/∗∗∗^ *p* < 0.001 and ^####/∗∗∗∗^ *p* < 0.0001.

Further histological evaluation of endometriotic tissues was conducted to assess fibrosis and inflammatory cell infiltration using H&E staining (Figure 7). The disease control group exhibited a significantly higher fibrosis score compared to the naïve control, indicating extensive stromal remodeling and chronic inflammation typical of advanced endometriotic lesions. Mice treated with the dydrogesterone–letrozole combination showed a reduction in fibrosis scores relative to the disease control group, suggesting a potential antifibrotic trend. This reduction appeared to follow a trend greater than that observed with dydrogesterone or letrozole monotherapy. Among the reference controls, leuprolide treatment resulted in a noticeable decline in fibrosis relative to the disease control, while dienogest failed to produce any notable improvement in fibrosis score (Figure 7).

## 4. Discussion

Treating endometriosis presents numerous challenges due to the complexity of the disease, which involves estrogen‐dependent lesion growth, immune dysregulation, and chronic inflammation [1–3]. Current therapeutic strategies for endometriosis focus on hormonal suppression to alleviate symptoms, inhibit lesion growth, and improve quality of life [27]. Progestins (dienogest, norethindrone acetate, etc.) are widely used as first‐line therapy due to their ability to induce decidualization and atrophy of ectopic endometrial tissue; however, their use may be limited by side effects including mood changes, weight gain, and irregular bleeding [7]. COCs are also commonly prescribed to suppress ovulation and stabilize hormonal fluctuations, though they may cause nausea, breast tenderness, and breakthrough bleeding [28].

In patients with an inadequate response or poor tolerance to first‐line treatments, management of endometriosis shifts to more advanced hormonal therapies. GnRH agonists (e.g., leuprolide and goserelin) and antagonists (e.g., elagolix) are used as second‐line options to induce a reversible hypoestrogenic state [6]. While effective in reducing pain and lesion burden, these agents are often associated with menopausal‐like side effects such as hot flashes, bone mineral density loss, vaginal dryness, and mood disturbances [6, 29]. The induced hypoestrogenic state by GnRH agonists also leads to impaired fertility during the treatment [30]. Consequently, there is a need for new treatments that effectively treat this multifactorial disease and address fertility issues.

Among progestins, dydrogesterone does not inhibit ovulation or compromise fertility, making it a valuable option for women with endometriosis [10]. Its favorable safety profile further distinguishes it as a promising choice compared to other progestins [31]. Dydrogesterone has demonstrated clinical efficacy as a monotherapy in alleviating endometriosis‐related symptoms, such as pelvic pain, and reducing the size of ectopic lesions. A meta‐analysis of 19 studies (1709 women) reported that dydrogesterone significantly improved pelvic pain and dysmenorrhea and lowered the occurrence of adverse events [10]. Clinical trial in Indian patients demonstrated a pronounced decrease in the size of endometrioma, endometriosis‐associated pelvic pain, serum VEGF levels, and significantly improved the quality of life [32]. Although dydrogesterone targets multiple aspects of endometriosis, it does not fully address the multifactorial nature of the disease, as it has shown limited effectiveness in suppressing local estrogen production [12].

Aromatase is a key enzyme involved in estrogen biosynthesis and is abnormally overexpressed in endometriotic tissues. Among the aromatase inhibitors, letrozole has demonstrated better estrogen suppression, and it effectively reduced endometrioma, pelvic pain, and dyspareunia in multiple clinical trials [33, 34]. Although letrozole has demonstrated efficacy in reducing endometriosis‐associated symptoms in fertile women (premenopausal), an aromatase inhibitor alone may induce ovarian folliculogenesis, and thus aromatase inhibitors are combined with a progestin, a COC, or a GnRH analog in order to prevent reflex increments in luteinizing hormone and follicle‐stimulating hormone [16, 35, 36]. A clinical trial involving 35 premenopausal women with rectovaginal endometriosis demonstrated that the combination of letrozole and norethindrone acetate resulted in a substantial decrease in endometrioma volume compared to either monotherapy [16]. Another small pilot study in 10 reproductive‐aged women demonstrated that a combination of letrozole, norethindrone acetate, calcium, and vitamin D for 6 months effectively reduced laparoscopically visible endometriotic lesions and decreased pelvic pain scores [36]. Similarly, a case study involving a postmenopausal woman reported enhanced efficacy of a combination of letrozole and MPA in reducing endometrioma volume [37].

Furthermore, studies have shown that letrozole in combination with progestins was well tolerated and offered a multitargeted approach to manage endometriosis [15]. The progestogenic effect of progestin (norethisterone acetate) counteracted the extreme estrogen deficiency caused by letrozole, and mitigated associated adverse effects such as hot flashes and osteoporosis [16]. Hence, the dydrogesterone–letrozole combination may represent a complementary therapeutic strategy for endometriosis by targeting both estrogen excess and progesterone resistance. Additionally, the dydrogesterone–letrozole combination may be associated with improved fertility outcomes in patients with endometriosis, since both drugs support ovulation in individual studies [10].

In the present study, we have evaluated the therapeutic effects of a dydrogesterone–letrozole combination in a preclinical mouse model of endometriosis and compared the outcomes relative to the disease control, with reference to standard treatments (dienogest and leuprolide). The doses of dydrogesterone (5 mg/kg) and letrozole (0.5 mg/kg) used in mice were calculated according to FDA guidelines and correspond to the maximum recommended oral daily doses in humans, which are approximately 20–30 mg for dydrogesterone and 2.5 mg for letrozole [21].

In our study, simultaneous daily oral administration of the dydrogesterone–letrozole combination in endometriosis was designed to ensure consistent overlap of pharmacodynamic effects. Dydrogesterone is approved for threatened or recurrent miscarriage, secondary amenorrhea, dysmenorrhea, and luteal phase support, and it is typically administered orally at 10–20 mg/day. Dydrogesterone has a short elimination half‐life of ∼5–7 h; however, its active metabolite, 20α‐dihydrodydrogesterone, has a half‐life of 14–17 h. Letrozole is approved for hormone receptor‐positive breast cancer in postmenopausal women at a dose of 2.5 mg/day and has a half‐life of ∼40 h. When administered together, dydrogesterone may provide progestogenic effects that counteract progesterone resistance, reduce inflammation, and support endometrial differentiation, while letrozole may suppress systemic and local estrogen synthesis that supports lesion growth. The dosing regimen, pharmacokinetics, and complementary pharmacodynamics of dydrogesterone and letrozole support their convenient once‐daily coadministration. Importantly, this regimen is supported by clinical evidence [17, 38]. Several randomized controlled trials have evaluated daily oral coadministration of dydrogesterone (10–20 mg) with letrozole (2.5 mg) for the treatment of endometriosis. These studies reported improved clinical outcomes compared with monotherapy, along with a favorable safety and tolerability profile [17, 38]. Therefore, our dosing strategy in mice aligns with the translational goals of the preclinical model and is consistent with existing clinical data.

Potential challenges of long‐term coadministration of dydrogesterone and letrozole include hypoestrogenic effects or tolerability issues. However, dydrogesterone’s ovulation‐sparing effect and progestogenic activity may reduce these risks. Importantly, existing clinical trials of letrozole combined with dydrogesterone [17, 38] or norethisterone acetate [16] in women with endometriosis did not report adverse effects such as follicular cysts, antral follicle count (AFC) reduction, or endometrioma enlargement, and overall tolerability was favorable. These findings support the translational potential of the dydrogesterone–letrozole combination.

Our results indicated trends toward reductions in the volume of endometriotic lesions with the dydrogesterone–letrozole combination relative to the disease control. Observed trends suggested numerically greater lesion reduction compared to individual treatment groups, though direct statistical comparisons between treatment arms were not performed. These trends may reflect additive or synergistic effects, potentially related to suppression of local estrogen synthesis by letrozole together with the anti‐inflammatory and antiproliferative properties attributed to dydrogesterone based on prior literature [10, 26]. Leuprolide was associated with a modest trend toward smaller lesions, whereas dienogest showed a trend toward reduced lesion volume, which appeared less pronounced than that observed with the dydrogesterone–letrozole combination.

Histopathological analysis suggested trends of improvement in stromal and glandular tissue scores, epithelial proliferation, and fibrosis with the dydrogesterone–letrozole combination compared to monotherapies. These trends were reflected in a decrease in the total histopathology score. The stromal component of ectopic lesions is known to contribute to chronic inflammation and fibrosis via altered cytokine production and progesterone resistance, while the glandular epithelium supports lesion persistence through estrogen‐driven proliferation and impaired apoptosis [39, 40]; therefore, the observed histological trends may be consistent with these previously described mechanisms, although they were not directly assessed in the present study. Letrozole was associated with trends toward reduced epithelial proliferation and fibrosis, whereas dydrogesterone may have contributed to stromal modulation, consistent with its known progestogenic activity. Overall, these observations suggest a dual‐target approach, combining hormonal modulation with anti‐inflammatory effects, with trends toward enhanced histopathological improvements relative to monotherapies. Further, both standard treatments (dienogest and leuprolide) showed less pronounced trends compared to the dydrogesterone–letrozole combination in reducing total histopathology scores.

PCNA is a well‐established marker for cellular proliferation. The dydrogesterone–letrozole combination was associated with trends of reduced PCNA‐positive cells relative to vehicle or disease control, suggesting a potential antiproliferative effect, with numerically lower PCNA expression than observed in other treatment groups. Leuprolide demonstrated trends toward antiproliferative effects, whereas dienogest appeared to exert modest effects relative to the disease control. These findings align with literature‐based hypotheses that suppressing aromatase activity (letrozole) combined with progestogenic support (dydrogesterone) may attenuate estrogen‐driven proliferation of ectopic tissue [16, 17].

VEGF plays a key role in the pathophysiology of endometriosis by promoting angiogenesis. Proinflammatory cytokines such as TNF‐α, IL‐6, and IL‐1β have been shown to induce NF‐κB activation, which in turn regulates VEGF expression [1, 28, 41]. In our study, the dydrogesterone–letrozole combination was associated with trends toward reduced VEGF, TNF‐α, and IL‐1β levels compared to the disease control, suggesting potential anti‐inflammatory and antiangiogenic effects. Monotherapies (dydrogesterone or letrozole) and reference drugs (dienogest and leuprolide) showed modest or nonsignificant trends. The dydrogesterone–letrozole combination also appeared to reduce tissue fibrosis, a hallmark of chronic endometriosis and a contributor to pelvic pain and adhesions. Leuprolide showed partial antifibrotic trends, whereas dienogest did not appear to reduce fibrosis, highlighting the limited impact of standard progestin therapy on fibrotic remodeling under the conditions tested.

Further, we have also evaluated the drug–drug interaction between dydrogesterone and letrozole in human hepatocytes. This study was conducted to assess whether coadministering the dydrogesterone–letrozole combination would alter their individual metabolism, which could potentially impact their effectiveness or toxicity profiles. Our results demonstrated that the metabolism of dydrogesterone or letrozole remained unaffected in the dydrogesterone–letrozole combination, indicating no potential drug–drug interaction (Table 1).

Collectively, these findings provide preclinical evidence that the dydrogesterone–letrozole combination is associated with measurable biological effects in a mouse model of endometriosis.

Most clinical studies evaluating the combination of progestins and letrozole have reported beneficial effects primarily related to symptomatic relief, including reductions in pelvic pain and improvements in quality of life [15]. However, these investigations have largely been observational in nature and have not thoroughly examined the underlying pathological mechanisms. In contrast, our study suggests trends toward reductions in lesion volume and improvements in histopathological features, including epithelial proliferation, fibrosis, inflammatory cytokine expression, and angiogenic marker levels. These observations provide preliminary mechanistic insight, consistent with existing literature, but direct assessments of progesterone resistance, estrogen suppression, stromal remodeling, or ovulatory function were not conducted. Future studies measuring hormone receptor expression, apoptosis markers, and ovulatory function will be required to confirm these mechanisms.

While the current study provides valuable insights, it has some limitations that warrant careful consideration. The experimental endometriosis model was established in nonmenstruating mice, which do not naturally develop endometriosis and therefore do not fully recapitulate the hormonal and immunological environment of the human disease. Unlike humans, mice do not undergo spontaneous menstrual shedding or retrograde menstruation, which are central to the initiation and cyclical progression of endometriosis in women. Consequently, lesion development in this model depends on surgical implantation and requires exogenous estrogen supplementation to sustain ectopic tissue survival. This creates a persistently hyperestrogenic state that differs from the fluctuating hormonal milieu characteristic of the human menstrual cycle. These hormonal discrepancies may alter immune activation, stromal–epithelial interactions, angiogenesis, and fibrosis pathways, potentially influencing both disease phenotype and therapeutic responses. Therefore, while the model is widely used and valuable for mechanistic and pharmacological studies, these physiological differences should be carefully considered when interpreting the translational relevance of the findings. Overall, the present work provides preclinical, proof‐of‐concept evidence generated in a mouse model, and confirmation of the therapeutic potential of the dydrogesterone–letrozole combination will require well‐designed clinical studies in humans.

## 5. Conclusion

The current study demonstrates that the dydrogesterone–letrozole combination was associated with measurable biological effects in a preclinical mouse model of endometriosis. Treatment with the dydrogesterone–letrozole combination was associated with trends toward reductions in endometriotic lesion volume, cellular proliferation, inflammatory cytokines, angiogenic markers, and tissue fibrosis relative to the disease control and reference treatments (dienogest and leuprolide). The observed trends may reflect complementary actions of dydrogesterone and letrozole, as monotherapies were generally associated with modest effects across the assessed endpoints. These findings provide exploratory, proof‐of‐concept evidence supporting further investigation of the dydrogesterone–lLetrozole combination. Extended‐duration preclinical studies are required to assess long‐term safety, efficacy, reproductive outcomes, and the potential for disease modification, which will be critical for designing future clinical studies to evaluate this therapeutic strategy for endometriosis.

## Funding

No funding was received for this research.

## Conflicts of Interest

The authors declare no conflicts of interest.

## Data Availability

The data that support the findings of this study are available from the corresponding author upon reasonable request.
